# Parallel Tagged Next-Generation Sequencing on Pooled Samples – A New Approach for Population Genetics in Ecology and Conservation

**DOI:** 10.1371/journal.pone.0061471

**Published:** 2013-04-18

**Authors:** Monika Zavodna, Catherine E. Grueber, Neil J. Gemmell

**Affiliations:** 1 Centre for Reproduction and Genomics, Department of Anatomy, University of Otago, Dunedin, New Zealand; 2 Department of Zoology, University of Otago, Dunedin, New Zealand; 3 Allan Wilson Centre for Molecular Ecology and Evolution, University of Otago, Dunedin, New Zealand; Monash University, Australia

## Abstract

Next-generation sequencing (NGS) on pooled samples has already been broadly applied in human medical diagnostics and plant and animal breeding. However, thus far it has been only sparingly employed in ecology and conservation, where it may serve as a useful diagnostic tool for rapid assessment of species genetic diversity and structure at the population level. Here we undertake a comprehensive evaluation of the accuracy, practicality and limitations of parallel tagged amplicon NGS on pooled population samples for estimating species population diversity and structure. We obtained *16S* and *Cyt b* data from 20 populations of *Leiopelma hochstetteri*, a frog species of conservation concern in New Zealand, using two approaches – parallel tagged NGS on pooled population samples and individual Sanger sequenced samples. Data from each approach were then used to estimate two standard population genetic parameters, nucleotide diversity (π) and population differentiation (F_ST_), that enable population genetic inference in a species conservation context. We found a positive correlation between our two approaches for population genetic estimates, showing that the pooled population NGS approach is a reliable, rapid and appropriate method for population genetic inference in an ecological and conservation context. Our experimental design also allowed us to identify both the strengths and weaknesses of the pooled population NGS approach and outline some guidelines and suggestions that might be considered when planning future projects.

## Introduction

Rapid improvements in next-generation sequencing (NGS) platforms, also called deep sequencing, have greatly increased the quantity of genetic data that can be affordably obtained in a relatively short time. These technologies have already found application in ecology, conservation and evolutionary biology: for whole genome sequencing of model and non-model organisms, transcriptomics, as screening tools to identify and validate new genetic markers and mutations, and as tools for high throughput molecular systematics (e.g. [1]–[6]).

An important feature of NGS is that it is equivalent to cloning DNA products derived from a single DNA molecule in a cell-free system and then sequencing a high number of these clones. This facet of the technology is of a particular interest in population genetics as it allows characterization of genetic variability of highly polymorphic and multi-copy genes, for which many different variants may co-occur within individuals, without cloning [7]. Moreover, samples can be barcoded/tagged and pooled (multiplexed) in a single run, allowing the sequencing of large number of samples in parallel, thus reducing cost of sequencing, while also increasing throughput. The barcodes/tags can be either directly included in the amplification primers [8] or ligated to DNA fragments after amplification prior to sequencing [9].

One major barrier to the widespread application of this technology in population genetic studies is the cost and time needed to tag the relatively large number of individual samples routinely required for such work, which may make the use of NGS approaches unaffordable for many. However, for many population genetic studies, especially those in conservation biology, human medical diagnostics or plant and animal breeding, the genetic information sought typically resides at the population level rather than at that of the individual. Thus, one way to overcome the significant cost of individual tagging is to pool individuals, for example, by population and use barcodes/tags to identify populations instead of individual samples [10], [11]. Such pooling approaches dramatically reduce the number of tags needed for a typical NGS experiment, providing a more cost-effective and rapid approach to determine the population genetic parameters, such as nucleotide diversity (π) and population differentiation (F_ST_) routinely sought.

Pooling, like other methods, is sensitive to sequencing errors and variation in DNA concentration among individuals in the pool. However, with appropriate experimental controls, rapid improvements in the quality and depth of sequencing now routinely obtained, and new efficient computational analysis tools, pooling approaches can lead to improved SNP discovery, better estimates of population allele frequencies and increased accuracy in population genetic inference [12].

In human medical diagnostics, rare variants of large effect have been recognized as conferring substantial risks for common human diseases and complex traits [13]. Sequencing large sample cohorts is therefore essential to discover the full spectrum of genetic variants and provide sufficient power to detect differences in allele frequencies between cases and controls. In such cases, the use of NGS on pooled samples has proven to reliably detect rare variants and estimate population allele frequencies [14]–[18]. Another field where application of NGS on pooled samples has proven to be very powerful is plant and animal breeding research programmes, where this approach has been used to characterize polymorphisms in candidate genes associated with critical traits (e.g. [19]–[21]).

However, NGS on pooled population samples has thus far been only sparingly employed in ecological [11], [22] and evolutionary [10] studies. Furthermore, the pooling approach and NGS should be of a particular interest in conservation biology, where it could have wide applicability for estimating population genetic parameters. These approaches would be well suited to addressing a variety of simple, but important questions typically asked by conservators, such as: “How much genetic diversity resides within a given species?”, “How is diversity distributed among the populations?” and/or “Are populations genetically differentiated?”, providing a basis for the identification of populations likely to be important for further conservation research and/or the management of that species [23]. Thus, we think the NGS pooled population approach might be viewed as an initial “triage” tool in the conservation genetics toolkit to determine further research and management decisions for a given species, rather than an ultimate population genetics approach for the species’ ecology and conservation.

Here we examined how accurate, reliable and appropriate parallel tagged amplicon NGS on pooled population samples is for estimating species population diversity and structure, and thus providing population genetic inference in species conservation context. Our study species was the endemic New Zealand frog *Leiopelma hochstetteri*, a species of conservation concern in New Zealand. Prior work by Fouquet et al. [24] documented strong genetic structure among *L. hochstetteri* populations based on individual Sanger sequence data from mitochondrial (*16S* and *Cyt b*) and nuclear (Tyrosinase) genes. Utilizing the same genomic DNA samples from the Fouquet et al. [24] study, we performed parallel tagged amplicon NGS on pooled population samples, estimated population genetic parameters and compared these with population genetic parameters derived from the individual Sanger sequence data. Thus, unlike the above-mentioned ecological studies, which did not or only partially validated pooling NGS results by individual Sanger sequencing [11], [22], our study provides a comprehensive evaluation of the accuracy, practicality and limitations of parallel tagged amplicon NGS on pooled population samples for estimating population genetic parameters. We explore both the strengths and weaknesses of this pooling approach and outline some guidelines and suggestions that might be considered when planning such projects.

## Materials and Methods

### Samples and Amplifications

Genomic DNA samples for 93 *Leiopelma hochstetteri* individuals, representing 20 New Zealand populations were obtained from a previous study [24]. The number of individuals per population ranged from 2 to 13 with a mean (± SE) of 4.65±0.54 and covered the full geographic range of this species. More details on *L. hochstetteri*’s biology, life history and populations sampled are provided in Fouquet et al. [24]. All work was undertaken under permits issued by the New Zealand Department of Conservation (Permit Number: NHS-05-06-12), and with the approval of the University of Canterbury Animal Ethics Committee (Permit Number: 2000-14).

In this study, two mitochondrial genes, *16 S* and *Cyt b*, were amplified by polymerase chain reaction (PCR). For *16 S*, we used primers 16F3Leio and 16RLeio, which amplify a 420-bp fragment [24]. For *Cyt b*, we used two combinations of primers: JB1F and CBLeioR, which amplify a 570-bp fragment, and JB1F and JB36R, which amplify a longer 830-bp fragment, encompassing the first fragment [24]. Thus, for each individual we amplified one *16S* and two overlapping *Cyt b* fragments. All amplification reactions were performed in a 25 µl total volume containing 10 ng of template DNA, 1× NH_4_ reaction buffer (16 mM (NH_4_)_2_SO_4_, 67 mM Tris-HCl pH 8.8, 0.01% stabilizer; supplied with BIOTAQ™ DNA Polymerase), 2 mM MgCl_2_, 400 nM of each primer, 200 µM of each dNTP and 1 Unit BIOTAQ™ DNA Polymerase (Bioline). PCR conditions were 94°C for 2 min followed by 35 cycles of 94°C for 30s, 52°C for 30s, 72°C for 45s, and a final extension at 72°C for 4 min. PCR products were verified on a 1% agarose gel stained with SYBR® Safe.

### Parallel Tagged Next-generation Sequencing on Pooled Population Samples

All amplified PCR products (amplicons) were purified using an Agencourt AMPure XP DNA purification kit following the manufacturer’s instructions (Beckman Coulter). DNA concentrations of purified amplicons were measured using a Quant-iT™ PicoGreen® dsDNA Assay kit (Invitrogen, Molecular Probes). Equimolar amounts of individual amplicons for both, *16 S* and *Cyt b* genes were pooled by population, creating 20 population-pools. These were barcoded (tagged) using a blunt-end ligation protocol [9]. For designing the barcodes, we used 8-bp long tags, with a minimum substitution distance = 3, selected from the supplementary material in [9]. The pooled, barcoded samples were sequenced on a 1/16 region of a GS FLX Titanium instrument (Roche Diagnostics).

### Data Analyses – pooled Population Samples Approach

The obtained NGS reads were initially examined using the Genome Sequencer FLX System Software version 2.3 and its SFF Tools commands (Roche Diagnostics). Only reads that contained complete population-specific barcodes with no mismatches were extracted for later analysis. Extracted reads were analyzed on a per-population basis and assessed for Phred quality scores and read length. The 20 Standard Flowgram Format (sff) files (one for each population) were further processed using CLC Genomics Workbench 4.9 (*CLC bio*). Reads were mapped to the *16S* and *Cyt b* reference sequences. Both references were generated as consensus sequences from the *L. hochstetteri 16S* and *Cyt b* sequences available in GenBank. The CLC mapping parameters were set to the following: mismatch, insertion and deletion cost = 2, length fraction = 0.75, similarity fraction = 0.9. This filtering included in the final mappings only reads, which had greater than 90% similarity with the reference in at least 75% of their read length. It is important to note that the CLC Genomics Workbench mapping parameters do not filter reads based on the read length per se, thus reads of various length may be included in the final mappings that are then used for the SNP detection. The criteria for SNP detection were set to minimum central and neighboring Phred quality score = 20, minimum coverage = 20 reads, and a minimum variant frequency was adjusted for each population based on the number of pooled individuals. For example, if the population consists of seven individuals, the frequency of a single copy variant/allele for a mitochondrial haplotype is expected to be approximately 14% (1 in 7). Low frequency variants/alleles may result from sequencing errors [7], thus to be conservative any SNP variants with a frequency less than 5%, approximately our lowest expected level of real haplotypes, were excluded from all populations.

One limitation of the current CLC Genomics Workbench analyses is that they provide only information on frequencies of variants at the individual SNPs among the reads, but not the phase (combination) of these variants among the multiple SNPs within the reads. Furthermore, because the final mappings do not typically include only full-length reads, the reconstruction of population haplotypes for both *16S* and *Cyt b* mitochondrial genes was inferred as follows: each population pool contained equimolar amounts of a known number of individuals, thus the frequencies of SNPs identified and calculated from our NGS data using CLC Genomics Workbench should approximate the frequency of those SNP variants in a given population. For example, if in a population-pool of seven individuals, detected frequency of variants C/T at a given SNP position of a haploid mtDNA gene is about 0.7/0.3, respectively, five individuals likely exhibit at this position variant C and two individuals exhibit variant T. Such inference allows reconstruction of population haplotypes, however given the current limitations of the analyses, the phasing of the variants at multiple polymorphic sites (SNPs) within an individual haplotype could not be automated and was therefore resolved by eye using longer reads in the final mappings. The haplotypes were then used in further population genetic analyses.

Estimates of population nucleotide diversity (π) and pairwise population differentiation (F_ST_) were calculated using DnaSP 5.10.01 [25] for *16S* haplotypes and *Cyt b* haplotypes separately to assess the possibility that differences in the accuracy of our approach are influenced by amplicon length. The pairwise F_ST_ values were calculated according to Hudson et al. [26] and based on the mean number of differences between different sequences sampled from the same population and sampled from two different populations. As such, the calculated pairwise F_ST_ values can be considered as estimates of similarity/dissimilarity between populations. Population genetic structure inferred from *16S* haplotypes and *Cyt b* haplotypes was also assessed using an analysis of molecular variance (AMOVA) with 1000 permutations, as implemented in the software Arlequin 3.5. [27]. AMOVA calculates analogs of Wright’s [28], [29] hierarchical *F*-statistics, designated as Φ-statistics.

### Data Analyses – Individually Sanger Sequenced Samples Approach

All individuals used in the pooling approach in this study had been previously sequenced by the conventional Sanger method for both the *16S* and *Cyt b* genes ([24]; the GenBank accession numbers FJ950428– FJ950532 and FJ950322– FJ950427 for *16S* and *Cyt b*, respectively). We retrieved the relevant sequences from the GenBank, grouped them by population and also analyzed these using CLC Genomics Workbench 4.9 (*CLC bio*) to eliminate software bias or error. The analyses were performed as above with the following modifications: under the function specific to Sanger sequence data, the grouped sequences were assembled to *16S* and *Cyt b* references using default parameters; the minimum coverage for SNP detection was set to two ( =  smallest population size); and minimum variant frequency was adjusted for each population based on the number of pooled individuals.

As in our pooled NGS approach, the polymorphic sites within and between populations and the identified SNP variants/alleles at these sites were used to reconstruct population haplotypes for *16S* and *Cyt b* mitochondrial genes. However, in this individual samples approach, the number of sequences/reads equals the number of individuals and therefore variant counts, rather than frequencies, were used. Furthermore, because of the full read through with Sanger sequencing and presence of only one sequence/read per individual, the combination of the variants at multiple polymorphic sites within an individual sequence could be unambiguously resolved.

Finally, estimates of population nucleotide diversity (π) and pairwise population differentiation (F_ST_) were calculated using DnaSP 5.10.01 software [25] and Φ-statistics using Arlequin 3.5 software [27] for *16S* haplotypes and *Cyt b* haplotypes separately in order to facilitate comparison between two approaches.

### Comparison of the Population Genetic Structure Estimates and Genetic Inference Obtained using Our Two Approaches

In order to assess how reliable and appropriate parallel tagged NGS on pooled population samples is for estimating population genetic parameters we compared the data derived from our pooled population sequencing approach to those obtained using the individual-based sequencing approach and evaluated their correlation. For each of the *16S* and *Cyt b* genes we compared the nucleotide diversity (π) estimates calculated for each population under the two approaches (i.e. *N* = 20 population estimates), as well as the estimates of pairwise population differentiation (F_ST_) under the two approaches (i.e. *N* = 190 pairwise comparisons between population-pairs). Our statistics allowed us to validate the utility of the pooled approach by addressing two main questions:

1. Were the estimates from the pooled-population approach equal to estimates from the individual-based approach? We built a linear regression between the values calculated under the two approaches and examined whether the confidence interval for the fitted slope contained our β = 1 expectation, which would indicate that two approaches provide identical values on average. Moreover, R2 for the linear regression was examined as an indication of the degree of similarity between the two approaches.
2. Do the two approaches permit us to make the same inference about population diversity and differentiation? We addressed this question by considering the qualitative guidelines for interpretation of both π and FST [30], where for each measure in turn the minimum value of 0 indicates no genetic diversity and no population differentiation, while the maximum values of 1 indicate high genetic diversity and population differentiation, respectively. In a conservation context, critical qualitative genetic inference is based on non-zero values of π and FST. In other words, one would want to know if populations are genetically diverse (π >0) and whether populations are genetically differentiated from each other (F_ST_ >0). To assess the performance of each approach in this context, we calculated the number of occurrences where the measure from one approach gave a 0 value when the other provided a non-0 value, as these would result in important differences in interpretation. For example, if π of a given population gives a non-0 value in one approach, i.e. indicating diversity among the pooled individuals, it may provoke further population genetic analysis on the individual level. Conversely, if for the same population π = 0 in the other approach, i.e. indicating no diversity among the pooled individuals, an argument against further population genetic analysis on the individual level might be made. Similarly, if pairwise F_ST_ of two populations equals 0 in one approach, i.e. indicating similarity and no differentiation between these populations, it might suggest these populations belong to one “evolutionary significant unit” (ESU) [31]; while if pairwise F_ST_ of the same two populations is non-0 value in the other approach, it might suggest these populations belong to different ESUs. Such differences in interpretations might ultimately lead to different conservation management and/or further research. Thus, we investigated in our data the proportion of populations (or population-pairs) for which a similar interpretation of the data would be obtained using either the pooling or individual sequencing approach, as these would ultimately guide similar further conservation and research decisions.

All statistical analyses were performed using functions available in the base package of R 2.12.2 [32].

## Results

The NGS yielded 34 495 reads that exactly matched one of the 20 population-specific barcodes (data deposited in the Dryad Repository: http://dx.doi.org/10.5061/dryad.f058c). Of these, 29 370 reads (85%) mapped to the *16S* reference (11 099 reads) or *Cyt b* reference (18 271 reads) under the mapping parameters described above. We obtained reads for all 20 populations and the number of reads per population ranged from 307 to 5054 with a mean (± SE) of 1469±281 reads, which reflected variability in number of individuals pooled per population.

Among *16S* reads across all populations in total, we identified the same number (*N* = 22) of polymorphic sites in both pooled and individual approaches. The number of SNPs within each population ranged from zero to seven in both approaches, with a mean of 2.6±0.36 in the individual Sanger sequencing approach and a mean of 2.5±0.36 in the pooled samples NGS approach, where two SNPs (each in a different population-pool) were not detected compared to individual approach. Overall, the number of identified SNPs for *16S* gene did not differ significantly between two approaches (Mann-Whitney test, U = 189, *P* = 0.77).

From *Cyt b* reads across all populations in total, we identified 60 polymorphic sites using the pooled samples NGS approach and 77 polymorphic sites using the individual Sanger sequencing approach. Among the polymorphic sites identified in the individual Sanger sequencing approach, eight were the result of ambiguous nucleotide codes in the sequences retrieved from GenBank. These sites were excluded from further analyses for the individual Sanger sequencing approach, giving a final dataset of 69 polymorphic sites. The number of SNPs within each population ranged from one to 19, mean 7.95±0.89 (SE), in the individual approach and zero to 14, mean 5.15±0.83, in the pooled approach. Across all populations we identified significantly fewer SNPs for *Cyt b* gene in the pooled samples approach compared to individual approach (Mann-Whitney test, U = 121, *P* = 0.03). This difference was further investigated (see below).

### Comparison of Population Genetic Structure Estimates and Genetic Inference Obtained using Our Two Approaches

#### *16S* haplotype data

**Figure 1:**
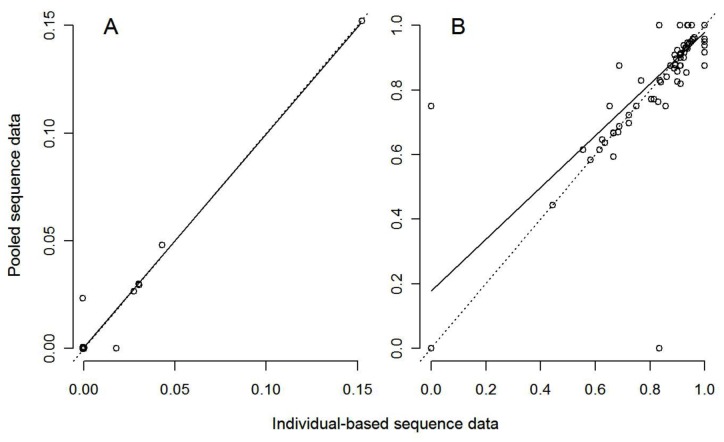
Relationship between pooled and individual approaches for estimating A) nucleotide diversity (π, *N = *20 populations shown) and B) pairwise population differentiation (F_ST,_ *N = *190 population-pairs shown) from *Leiopelma hochstetteri 16S* mtDNA data. The dotted lines are the expected relationship where β = 1 (i.e. the two approaches give identical estimates), the solid lines are the observed linear regression (Table 1).

Based on *16S* data, both pooled NGS and individual Sanger sequencing approaches revealed strong and significant genetic structure among *L. hochstetteri* populations (overall Φ_ST_ = 0.917 and 0.921, respectively; both *P*<0.0001). The linear regression models for both nucleotide diversity π and pairwise population differentiation F_ST_ showed positive correlations between approaches (Table 1, Fig. 1). For π, the 95% confidence interval for the regression included 1, indicating that overall the two approaches gave generally equivalent values (Table 1, Fig. 1A). For F_ST_, the regression slope was marginally, albeit significantly shallower (Table 1) suggesting that, although values from the two approaches were strongly correlated, they were not identical. The F_ST_ estimates obtained from the pooled-population approach were slightly overestimated compared to the individual-sequence approach (Fig. 1B).

**Table 1:** Linear regression model summary statistics for comparison of two approaches (pooled and individual) based on nucleotide diversity (π) and pairwise population differentiation (F_ST_) estimates from *Leiopelma hochstetteri 16S* and 830-bp *Cyt b* data.

Statistics	N	Intercept (SE)	Slope β (95% CI)	R^2^
***16S***				
π	20	0.001 (0.002)	0.989 (0.900–1.078)	0.96
F_ST_	190	0.178 (0.034)	0.801 (0.729–0.874)	0.71
***Cyt b***				
π	20	0.002 (0.004)	0.640 (0.228–1.051)	0.34
F_ST_	190	0.378 (0.060)	0.597 (0.470–0.725)	0.31

N: number of populations (π) or population-pairs (F_ST_).

To further evaluate the accuracy of the pooled approach in an ecological context, we calculated the proportion of cases for which the same interpretation of the data would be inferred using either approach. Based on the estimates from *16S* haplotype data, we found that the same interpretation of the results would be inferred from the two approaches in 18 out of 20 (90%) and in 187 out of 190 (98%) cases for π and F_ST,_ respectively (Fig. 1). In other words, in only two out of 20 and three out of 190 cases, for π and F_ST,_ respectively, the measure from one approach gave a 0 value when the other provided a non-0 value, which would result in important differences in interpretation of whether populations are diverse or not and whether they are differentiated or not.

#### *Cyt b* haplotype data

**Figure 2:**
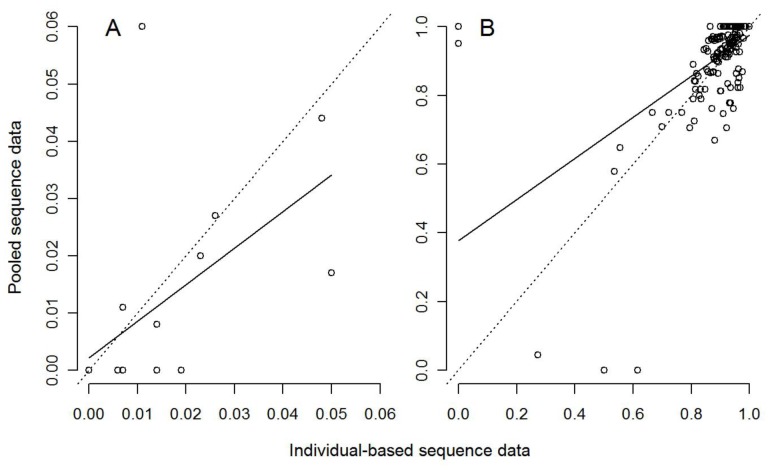
Relationship between pooled and individual approaches for estimating A) nucleotide diversity (π, *N = *20 populations shown) and B) pairwise population differentiation (F_ST,_ *N = *190 population-pairs shown) from *Leiopelma hochstetteri* 830-bp *Cyt b* mtDNA data. The dotted lines are the expected relationship where β = 1 (i.e. the two approaches give identical estimates), the solid lines are the observed linear regression (Table 1).

Strong and significant genetic structure among *L. hochstetteri* populations was also detected from *Cyt b* data with both pooled NGS and individual Sanger sequencing approaches (overall Φ_ST_ = 0.919 and 0.920, respectively; both *P*<0.0001). The linear regression model showed a positive relationship between the two approaches for each of π and F_ST_ (Table 1, Fig. 2). For π, the 95% confidence interval for the regression included our β = 1 expectation, however the interval was broad (Table 1) suggesting that although the values from two approaches were equivalent overall, there was high variability in degree of similarity between two approaches among the population-pools (Fig. 2A). For F_ST_, the regression slope was significantly shallower than our β = 1 expectation (Table 1) indicating that although values from the two approaches were positively related, they were not equivalent: F_ST_ estimates obtained from the pooled-population approach were overestimated compared to individual approach (Fig. 2B). Nevertheless, in an ecological context, the same conclusion about whether the population is diverse or not, and whether populations are differentiated or not, would be inferred in 16 out of 20 (80%) and in 186 out of 190 (98%) cases, respectively, from either approach (Fig. 2).

**Figure 3:**
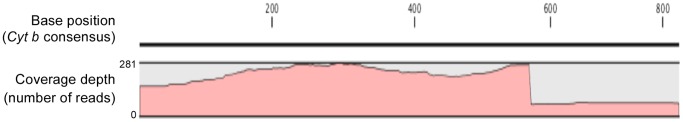
Example of a coverage plot for *Cyt b* mapping generated by CLC genomics workbench v 4.9. Data shown represent reads from one population-pool (*N* = 6 individuals).

**Figure 4:**
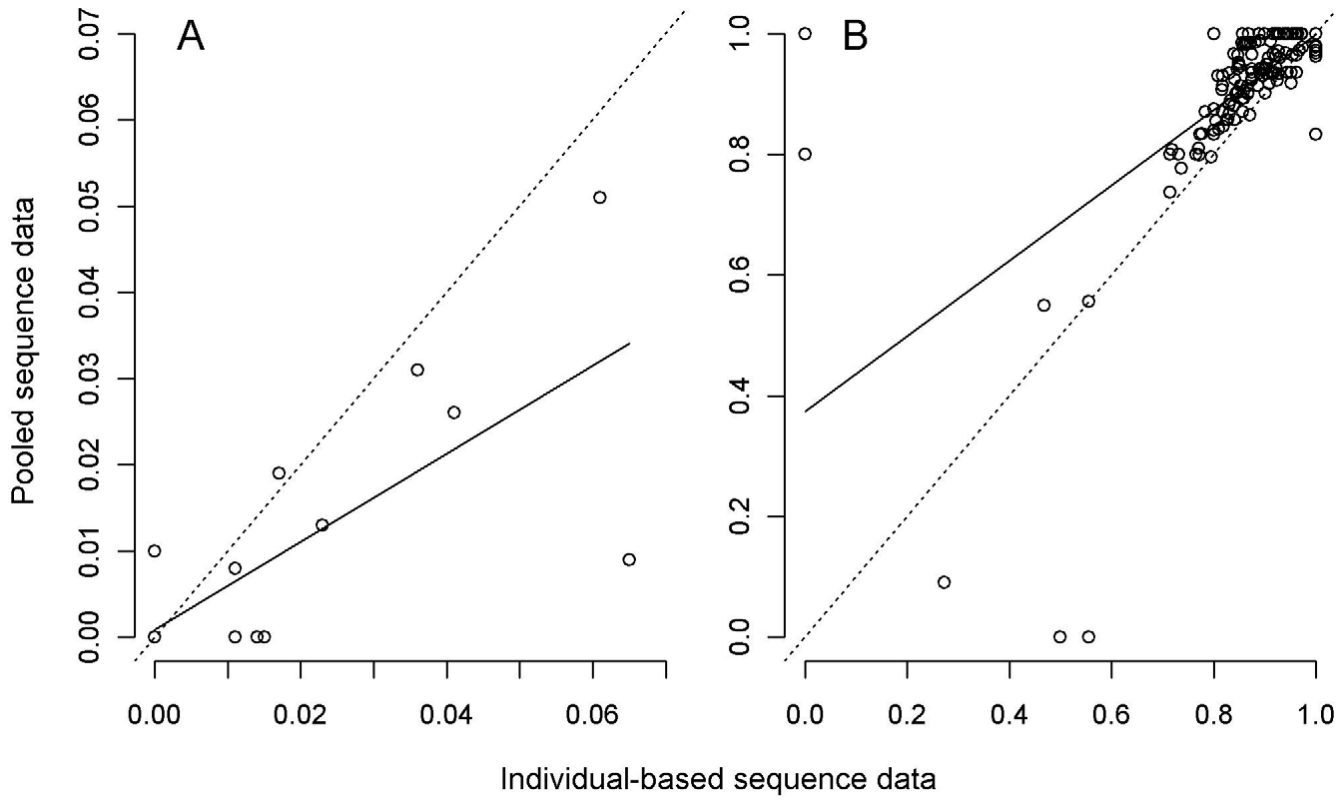
Relationship between pooled and individual approaches for estimating A) nucleotide diversity (π, *N = *20 populations shown) and B) pairwise population differentiation (F_ST,_ *N = *190 population-pairs shown) from *Leiopelma hochstetteri* 570-bp *Cyt b* mtDNA data. The dotted lines are the expected relationship where β = 1 (i.e. the two approaches give identical estimates), the solid lines are the observed linear regression (Table 2).

At first glance then, the estimates obtained for *Cyt b* using the pooled-population NGS approach showed a lower concordance with those derived from individual Sanger sequencing approach than those obtained for *16S*. We therefore sought to investigate the basis of this difference between the two genes and found that a number of *Cyt b* SNPs were undetected in the data obtained from our pooled population samples approach due to uneven read coverage across the fragment. Specifically, in the majority of the population-pools, much lower coverage was observed in the region spanning 500–830-bp (Fig. 3), likely a result of pooling and sequencing two overlapping *Cyt b* amplicons of different length (570-bp and 830-bp) for each individual. This amplification and sequencing approach meant the first ∼500-bp of the gene was represented by reads from both amplicons, while the region 500-bp+ was represented by reads from only one amplicon, resulting in uneven coverage across the longer-length fragment. In addition, both amplicons exceeded the sequencing instrument’s modal read length in our experiment (i.e. 477-bp on the GS FLX instrument we employed in 2011), and so the “forward” and “reverse” reads in our dataset further increased the coverage unevenness due to reads’ overlapping and/or not reaching entire amplicon length. Although undetected SNPs appeared across the entire *Cyt b* fragment, the overall number of SNPs detected in the first 500-bp did not differ significantly between our pooled NGS approach and the individual Sanger approach (Mann-Whitney test, U = 156, *P* = 0.23). However, the number of SNPs detected in the region spanning 500–830-bp differed significantly between our two approaches (Mann-Whitney test, U = 117.5, *P* = 0.02). A linear regression using π and F_ST_ derived from the first 570-bp of *Cyt b* data showed an improved correlation (increased R^2^) between the two approaches compared to our original analysis based on the whole fragment (Table 2, Fig. 4). To further understand the cause of discrepancies in SNP detection for our *Cyt b* data between the two approaches, we scrutinized the NGS population-pool mappings by eye. We found that over 50% of undetected SNPs across all populations were in fact present in at least two reads, but were not called in our analysis due to insufficient coverage or because they lay within a homopolymer region (Table S1). Pyrosequencing, which underpins the GS FLX platform, has proven difficulties in accurately sequencing homopolymers [33] and therefore analysis settings might exclude these regions from the final analysis. If we include SNPs that were observed by eye, but “not detected” in our *Cyt b* NGS data set analysis, the overall difference in number of SNPs detected between the two approaches is not statistically significant (Mann-Whitney test, U = 159, *P* = 0.27). These observations support our suggestion that lower coverage in the 500-bp+ region of the amplicon, compared to the rest of the *Cyt b* fragment, significantly affected overall efficacy of the pooling NGS approach relative to the individual Sanger sequencing approach for this gene. Interestingly, we also found that some polymorphic sites detected in the individual Sanger sequencing approach did not appear to be polymorphic despite high coverage using pooled samples NGS approach (Table S1). These cases were either rare (occurring in only one position in one population-pool), possibly the result of liquid handling errors leading to unequimolar pools, or appeared in the same position in multiple population-pools, indicating possible sequencing errors either in the individual Sanger sequencing or NGS.

**Table 2:** Linear regression model summary statistics for comparison of our two approaches (pooled and individual) based on nucleotide diversity (π) and pairwise population differentiation (F_ST_) estimates from *Leiopelma hochstetteri* 570-bp *Cyt b* data.

Statistics	N	Intercept(SE)	Slope β(95% CI)	R^2^
***Cyt b***				
π	20	0.001(0.003)	0.511(0.312−0.711)	0.58
F_ST_	190	0.374(0.050)	0.623(0.515−0.732)	0.40

N: number of populations (π) or population-pairs (F_ST_).

Overall, undetected *Cyt b* SNPs in the pooled population approach led to an underestimation of the population nucleotide diversity in some of the population-pools (Fig. 2A) and consequently overestimated pairwise population differentiation (Fig. 2B), relative to individual-based approach. This difference in the performance of NGS on population pools between *16S* and *Cyt b* is attributed to a key difference in the experimental design, that being the difference in target amplicon length versus the instrument’s modal read length and the number of amplicons sequenced per individual. This issue can readily be remedied (see below), and is not a fundamental flaw with the basic approach.

## Discussion

We have evaluated the accuracy and feasibility of a new approach for population genetics in an ecological and conservation context – parallel tagged amplicon NGS on pooled population samples. Our study used *16S* and *Cyt b* mitochondrial amplicons from *Leiopelma hochstetteri* individuals sampled from across their range to estimate the population genetic parameters and inference under two approaches – pooled population samples sequenced by NGS and individual samples sequenced using traditional Sanger sequencing. In contrast to those few ecological studies that have previously utilized pooled population NGS [11], [22], this dual approach enabled us to compare and validate the results of the pooling approach with individual sequencing applied to the entire data set. In addition we were able to examine amplicon-specific effects for our *16S* and *Cyt b* data separately, which enabled us to consider how differences in amplicon length and the number of amplicons obtained per individual affected the accuracy of our population genetic estimates and the inference we could make from those data. We found that amplicon length, in particular, caused significant differences in the overall quality of the results, which highlights a key limitation of the current technology.

For our *16S* work, only one amplicon per individual was present in each pool and its length of 420-bp was well within the GS FLX instrument’s modal read length (477-bp). Consequently, we obtained even NGS coverage across the entire amplicon for all pools and detected the same polymorphisms in our pooling approach as we did in the individual approach, resulting in high positive association for population genetic estimates between two approaches (Fig. 1, Table 1). Hence, under these conditions, the pooling approach has proven to be a reliable, rapid and appropriate method for population genetic inference in ecological and conservation context.

On the other hand, for *Cyt b*, we had two overlapping amplicons (570-bp and 830-bp) per individual in each population pool, both of which exceed NGS instrument’s modal read length (477-bp in our experiment using GS FLX in 2011). This resulted in undetected polymorphic sites between 500–830-bp in most of our NGS population-pools. Examination of alignments by eye suggested that many of these differences were due to insufficient sequence coverage for this part of the amplicon (Fig. 3), which in turn, led to an underestimation of genetic diversity in some of the populations we assessed using our NGS pooling approach (Fig. 2).

One possible issue resulting in the observed differences between *16S* and *Cyt b* might be a consequence of the different levels of within-population diversity identified from these two mitochondrial genes (Fig. 1A and 2A). A large number of populations exhibited no within-population variation (Fig. 1A) in *16S* gene, which is noteworthy because NGS of pooled population samples may be more accurate when estimating an allele frequency at a fixed locus than at a polymorphic locus. This is because at a fixed locus, there will be no errors in allele frequency estimates arising from quantitation or pipetting DNA to prepare the pooled samples, whereas the liquid handling errors might be a potential source of error at polymorphic loci. To investigate the influence of populations with π = 0 and population-pairs with F_ST_ = 0, we performed also linear regression analysis with these “zero” data removed for both *16S* and *Cyt b* genes. Not surprisingly, the positive association (R^2^) decreased, however only slightly (Table S2), indicating that low within-population variation had minimal effect on the differences observed between two mitochondrial genes. This suggests that NGS pooling approach is also a reliable and appropriate method for population genetic inference for species with higher intra-population diversity.

This experiment therefore provides crucial insights into the criteria needed to achieve high accuracy with the NGS pooling approach in population genetic studies and ultimately its practical utility. First, the length of the amplicons relative to the NGS instrument read length should be considered. We recommend that the total PCR product length be designed so that it lies well within the instrument’s read length, ensuring that the modal length of the reads then corresponds to length of the entire amplicon. This will facilitate more accurate downstream analysis not only for detection of polymorphisms, such as SNPs or indels, but also for resolving phase of the variants at linked polymorphic sites, as for example, only full-length-amplicon reads could be selected for downstream analysis. However, this recommendation does not necessarily preclude sequencing of longer amplicons. Such amplicons can be, for example, fragmented by nebulization or enzymatic digestion with fragmentase to achieve appropriate length of the fragments prior tagging and sequencing (e.g. [9]). With ongoing rapid increase in read lengths being achieved across many NGS platforms, read-length issues may become less of a concern in the near future.

Secondly, the number of individuals to be pooled should be considered, so that rare variants can be adequately detected and distinguished from sequencing errors [15]. This issue is related to sequencing depth (coverage), as the pooling approach becomes more efficient when coverage is high and the required minimum number of reads for allele calling is low [12]. For pools of large number of individuals, the sequencing error rate is likely to be close to, or even higher than the minor allele frequency (MAF, [17]). In such cases, it can be difficult to distinguish a true rare variant from a sequencing error. Wang et al [17] showed that empirical sequencing error rates were close to those expected based on quality Phred score. Thus, use of only reads with a high Phred score (>20) can reduce the negative effects of the sequencing errors. Empirical large-scale studies that have evaluated the accuracy of the pooling approach with respect to MAF have been conducted only in human disease diagnostics and genetics. These studies recommended a pool size such that MAFs are present at minimum of 0.5–1.2% [15], [16]. In our study, the MAF in the largest pool was estimated at over 5%, which ensured confident detection of rare variants. While such guidelines are useful, the massive read depths now being obtained on some NGS platforms may enable pool sizes to increase further, without increasing error rates due to sequencing artifacts.

The main result from our study, that NGS of DNA pools provides a reliable and appropriate approach for genome-wide allele frequency estimates, is supported by several recent studies in other contexts, in which the correlation between NGS of pools and individual genotyping has been shown to be high (r = 0.67–0.99) [15], [17], [34]. However, these prior studies compared allele frequencies, while we based our comparisons on the estimates of nucleotide diversity and population differentiation, as these parameters are more relevant to population genetics as applied in ecology and conservation. The strong correlation in our results between the pooled and individual approaches for our *16S* data supports the contention that NGS on pooled population samples is an accurate and reliable approach for estimating population genetic parameters in ecological population genetic studies.

From an ecological and conservation view point, our evaluation of the qualitative genetic inference obtained with both datasets suggests that pooling is also a rapid and feasible approach for assessing the genetic diversity and population genetic structure of a species. The same genetic interpretation was concluded from our NGS pooling strategy versus our individual-based approach in over 90% of cases for *16S* and over 80% of cases for the *Cyt b* data. Closer investigation of “incorrect” interpretations (i.e. those where one approach gave a 0 value when the other provided a non-0 value; Fig. 1 and Fig. 2) revealed that these were caused by a lower quality of NGS data at the given polymorphic sites, where true variants were filtered out by our analysis settings. This means that under less conservative parameter settings our analysis may have detected these variants, but at the same time would have increased the rate of false detection of spurious variants. We therefore anticipate that continuous improvements of the sequencing platforms and computational tools will further reduce any discrepancies.

The NGS on pooled population samples approach should be considered as a first and rapid, but not ultimate, step for assessing the population genetic parameters in the ecological context. To that end, we considered interpretations as “incorrect” only where one approach gave a 0 value when the other provided a non-0 value for π and F_ST_, as these might lead to critically different conservation management and/or further research actions as described above. However, subtle differences in non-0 π and F_ST_ values may result in similar subsequent actions (e.g. further genetic analysis on an individual level) depending on the question being addressed and therefore we did not consider these to be critical distinctions here.

We explored further possible outcomes of our NGS pooled population samples data for both *16S* and *Cyt b* using phylogenetic and molecular evolutionary analyses with MEGA version 4 [35]. Of the 13 clades revealed in Fouquet et al. [24] based on a concatenated sequence containing both *16S* and *Cyt b* sequence, we identified 10 of the same clades from our pooled NGS *16S* data and 12 of the same clades from our pooled NGS *Cyt b* data (data not shown). Note that our dataset consisted of 20 populations compared to 21 populations in the study of Fouquet et al. [24], where the unsampled population represented an independent clade in the previous study. Thus, our results suggest a high correlation between the NGS pooling approach and individual Sanger sequencing approach.

In summary, the parallel tagged amplicon NGS on pooled population samples is a rapid approach for estimating population genetic parameters in an ecological context. This approach provides reliable genetic information that has particular utility where baseline population genetic data are needed to identify species at risk and inform management plans [24], [36]. While the approach might be used to provide data for species conservation management directly, its best use may be for initial identification of populations, from which further validation by individual genotyping can be pursued as deemed necessary. Deep (NGS) sequencing of pools may serve as a useful diagnostic/screening tool for rapid assessment of species genetic structure at the population level, likely with significant utility in conservation threat assessment and management.

## Supporting Information

Table S1 Summary of all *Cyt b* polymorphic sites within population-pools. The SNP positions are given in bp. Y and N indicate whether given SNP, as identified in individual Sanger sequence approach, was or was not detected, respectively, in our pooled population NGS approach. Coverage is given in number of reads per mapping as analyzed in CLC genomics workbench prior to SNP detection step.
(PDF)

Table S2 Linear regression model summary statistics for comparison of two approaches (pooled and individual) based on nucleotide diversity (π) and pairwise population differentiation (FST) estimates from only “non-zero” values of *Leiopelma hochstetteri 16S* and 830-bp *Cyt b* data.
(PDF)
