# Supplementary material for: Parallel Tagged Next-Generation Sequencing on Pooled Samples – A New Approach for Population Genetics in Ecology and Conservation
Source: PLoS One. 2013 Apr 18;8(4):e61471. doi: 10.1371/journal.pone.0061471 (PMC3630221; doi:10.1371/journal.pone.0061471)
Supplement: Table S1 — Summary of all Cyt b polymorphic sites within population-pools. The SNP positions are given in bp. Y and N indicate whether given SNP, as identified in individual Sanger sequence approach, was or was not detected, respectively, in our pooled population NGS approach. Coverage is given in number of reads per mapping as analyzed in CLC genomics workbench prior to SNP detection step. (PDF) [file pone.0061471.s001.pdf]

Table S1

Summary of all *Cyt b* polymorphic sites within population-pools. The SNP positions are given in bp. Y and N indicate whether given SNP, as identified in individual Sanger sequence approach, was or was not detected, respectively, in our pooled population NGS approach. Coverage is given in number of reads per mapping as analyzed in CLC genomics workbench prior to SNP detection step.

Population-Pool	SNP position	Detected in NGS (Y/N)	Coverage (number of reads)	Comment
1	330	Y	468	
	461	Y	368	
	579	Y	35	
	653	Y	41	
	761	Y	41	
	770	Y	40	
2	41	N	488	
	47	N	507	
	57	Y	511	
	79	N	556	
	255	Y	954	
	284	Y	936	
	316	Y	938	
	330	Y	918	
	331	N	917	
	356	Y	884	
	389	Y	806	
	458	Y	685	
	713	Y	38	
3	17	Y	158	
	41	N	160	
	131	N	205	
	152	Y	229	
	176	Y	241	
	260	Y	267	
	263	Y	267	
	284	Y	269	
	317	Y	269	
	326	Y	259	
	353	N	244	present, but homopolymer
	470	N	211	
	482	N	217	
	515	Y	233	
	575	Y	63	
	636	Y	68	
	698	Y	71	
	740	Y	71	
	778	Y	71	

Table S1 *continued*

4	47	Y	178	
	57	Y	185	
	144	Y	268	
	199	Y	307	
	356	Y	334	
	759	N	5	present, but low coverage
	770	N	5	present, but low coverage
5	79	N	870	
	89	Y	878	
	245	Y	1299	
	335	N	1251	
	573	Y	116	
	594	N	120	present, but homopolymer
	627	Y	127	
	650	Y	137	
	720	Y	136	
6	41	Y	486	
	79	N	487	
	113	Y	485	
	152	Y	486	
	326	Y	269	
	330	Y	270	
	636	N	17	present, but low coverage
	740	Y	27	
	774	Y	27	
	782	Y	27	
	800	Y	27	
	809	Y	27	
7	41	N	180	
	356	Y	203	
	401	Y	213	
	579	N	5	present, but low coverage
	650	N	8	present, but low coverage
	653	N	8	low coverage
8	57	Y	88	
	356	Y	111	
	594	N	4	present, but low coverage
	650	N	3	low covergae
	744	N	2	present, but low coverage
9	79	N	1249	
	182	Y	739	
10	255	Y	65	
	305	N	93	present, but homopolymer
	330	N	111	present, but homopolymer
	356	Y	112	
	389	Y	114	
	458	Y	117	
	699	N	25	homopolymer

Table S1 *continued*

11	41	N	201	
	86	Y	201	
	158	N	208	present, but homopolymer
	263	N	93	present, but homopolymer
	335	Y	198	
	356	Y	199	
	515	Y	231	
	608	N	7	low coverage
	653	N	14	present, but low coverage
	693	N	16	low coverage
	778	N	15	low coverage
12	89	Y	127	
	573	N	7	present, but low coverage
	627	N	8	present, but low coverage
	650	N	13	present, but low coverage
	720	N	13	present, but low coverage
13	41	Y	260	
	152	Y	264	
	326	Y	196	
	330	Y	198	
	636	N	27	present, but low coverage
	681	N	38	present, but low coverage
	740	Y	37	
	774	Y	37	
	782	Y	37	
	800	Y	37	
	809	Y	37	
14	41	N	56	
	330	N	24	present, but low coverage
	461	Y	40	
	579	N	6	present, but low coverage
	638	N	17	present, but low coverage
	653	N	17	present, but low coverage
	761	N	16	present, but low coverage
	770	N	16	present, but low coverage
15	41	N	1279	
	62	Y	1365	
	137	Y	1850	
	222	Y	2183	
	272	Y	2085	
	456	Y	1733	
	543	Y	1947	
	599	Y	242	
	636	Y	277	
16	41	N	465	
	330	Y	894	
	461	Y	736	
	516	Y	780	
	579	Y	50	
	653	Y	65	
	761	Y	65	
	770	Y	65	
17	79	N	480	

Table S1 *continued*

18	50	Y	440	
	356	Y	846	
	650	N	42	present, but homopolymer
	653	Y	42	
	728	N	41	homopolymer
	732	Y	41	
19	86	Y	195	
	89	Y	195	
	573	N	8	present, but low coverage
	627	N	11	present, but low coverage
	650	N	11	present, but low coverage
	720	N	11	present, but low coverage
20	255	Y	354	
	326	Y	346	
	330	Y	341	
	336	Y	339	
	356	Y	335	
	389	Y	318	
	458	Y	272	
	803	Y	42	
