# Supplementary material for: Parallel Tagged Next-Generation Sequencing on Pooled Samples – A New Approach for Population Genetics in Ecology and Conservation
Source: PLoS One. 2013 Apr 18;8(4):e61471. doi: 10.1371/journal.pone.0061471 (PMC3630221; doi:10.1371/journal.pone.0061471)
Supplement: Table S2 — Linear regression model summary statistics for comparison of two approaches (pooled and individual) based on nucleotide diversity (π) and pairwise population differentiation (FST) estimates from only “non-zero” values of Leiopelma hochstetteri 16S and 830-bp Cyt b data. (PDF) [file pone.0061471.s002.pdf]

Table S2

Linear regression model summary statistics for comparison of two approaches (pooled and individual) based on nucleotide diversity (π) and pairwise population differentiation (F_{ST}) estimates from only "non-zero" values of *Leiopelma hochstetteri 16S* and 830-bp *Cyt b* data.

Statistics	N	Intercept (SE)	Slope β (95% CI)	R^2
16S				
π	7	0.002 (0.007)	0.964 (0.756 - 1.172)	0.94
F_{ST}	186	0.330 (0.043)	0.638 (0.546 - 0.731)	0.50
Cyt b				
π	11	0.007 (0.010)	0.475 (-0.301 - 1.252)	0.14
F_{ST}	190	0.378 (0.060)	0.597 (0.470 - 0.725)	0.31

N: number of populations (π) or population-pairs (F_{ST})
